# Construction and validation of nomogram model for high-risk early warning of medical complaints based on occupational characteristics and workload of medical staff

**DOI:** 10.3389/fpubh.2026.1816281

**Published:** 2026-05-21

**Authors:** Jiayi Huang, Tianyu Gou, Yang Cui, Fang Yang, Jinfeng Zhang

**Affiliations:** 1 Department of Humanities and Social Sciences, Harbin Medical University, Harbin, Heilongjiang, China; 2 Medical Records Department, The Fourth Affiliated Hospital of Harbin Medical University, Harbin, Heilongjiang, China; 3 Medical Department, The Fourth Affiliated Hospital of Harbin Medical University, Harbin, Heilongjiang, China; 4 Department of Nutrition and Food Hygiene, School of Public Health, Harbin Medical University, Harbin, China; 5 Harbin Medical University, Harbin, Heilongjiang, China; 6 Nursing Teaching and Research Department, The Fourth Affiliated Hospital of Harbin Medical University, Harbin, Heilongjiang, China

**Keywords:** burnout, healthcare professionals, legal awareness, medical dispute, nomogram, patient safety

## Abstract

**Background:**

The escalation of medical disputes and patient complaints represents a critical challenge to healthcare systems globally, particularly in high-volume tertiary hospitals in China. While patient-related risk factors are well-documented, the contribution of healthcare professionals' (HCPs) occupational characteristics, burnout levels, and legal awareness to dispute risk remains under-explored. This study aims to identify these provider-side risk factors and develop a predictive nomogram to quantify the risk of medical complaints.

**Methods:**

A single-center, cross-sectional retrospective study was conducted at the Fourth Affiliated Hospital of Harbin Medical University between May 2024 and August 2025. A total of 406 HCPs (187 physicians and 219 nurses) from high-risk departments (e.g., Neurosurgery, Orthopedics, Emergency Surgery) participated. Data on demographics, workload, Maslach Burnout Inventory (MBI) scores, defensive medical behaviors, and frequency of legal training were collected. The Least Absolute Shrinkage and Selection Operator (LASSO) regression was employed to screen variables, followed by multivariate logistic regression to identify independent predictors. A nomogram was constructed based on the final model and validated using the Concordance Index (C-index), calibration plots, and Decision Curve Analysis (DCA).

**Results:**

Of the 406 participants, 78 (19.2%) reported experiencing formal medical complaints in the past year. Multivariate analysis revealed that high emotional exhaustion (OR = 1.85, 95% CI: 1.23–2.78), depersonalization (OR = 2.10, 95% CI: 1.45–3.05), excessive night shifts (>5/month) (OR = 1.65, 95% CI: 1.12–2.43), and low frequency of legal training (0–1 time/year) (OR = 2.45, 95% CI: 1.56–3.89) were independent risk factors for complaints. Interestingly, defensive medical behaviors showed a U-shaped association. The developed nomogram demonstrated excellent discrimination with a C-index of 0.82 (95% CI: 0.76–0.88). Calibration curves showed good agreement between predicted and observed probabilities, and DCA confirmed the clinical utility of the model.

**Conclusion:**

The risk of medical complaints is significantly associated with HCP burnout, workload, and legal literacy. The proposed nomogram serves as an effective screening tool for hospital management to identify high-risk staff. Interventions focusing on psychological support and practical legal education are essential for mitigating medicolegal risks.

## 1 Introduction

Patient safety and the mitigation of medical disputes are central pillars of healthcare quality management. In recent decades, the incidence of medical malpractice claims and formal patient complaints has risen sharply worldwide, creating a “medicolegal crisis” that affects both developed and developing nations (1). In China, the tension in doctor-patient relationships has become particularly acute, often manifesting as disruptive incidents (e.g., “Yi Nao” or medical disturbance) or prolonged litigation, which imposes a heavy financial burden on hospitals and severe psychological trauma on healthcare professionals (HCPs) (2, 3). A systematic review indicated that a significant portion of adverse hospital events evolve into disputes not merely due to clinical errors, but due to communication failures and unmet expectations (1).

Traditionally, research on medical dispute risk factors has predominantly focused on patient characteristics (e.g., disease severity, age, socioeconomic status) or systemic factors (e.g., hospital infrastructure, staffing ratios) (4, 5). While predictive machine learning models have been successfully utilized for predicting medical disputes from a systemic and hospital-level perspective (3), the role of the individual service provider—the physician or nurse—has been relatively underemphasized in predictive modeling. HCPs operate under immense pressure, often characterized by long working hours, sleep deprivation, and high cognitive load. These occupational stressors are known precursors to burnout, a syndrome conceptualized as resulting from chronic workplace stress that has not been successfully managed (6, 7). Burnout, particularly its dimensions of emotional exhaustion and depersonalization, has been empirically linked to suboptimal patient care and increased medical errors (8), as well as to reduced empathy and impaired recognition of patients' emotional expressions (9), thereby potentially precipitating complaints. Yet, a quantitative tool that translates these psychometric properties into a tangible risk score for disputes is currently lacking.

Furthermore, the legal dimension of medical practice cannot be ignored. In the face of increasing litigation risk, “Defensive Medicine”—the practice of ordering tests, procedures, or visits primarily to reduce liability exposure rather than for patient benefit—has become prevalent (10). While intended as a shield, defensive medicine may paradoxically increase dispute risks by inflating costs and subjecting patients to unnecessary interventions, thereby eroding trust and potentially fostering a climate ripe for more litigation (11). From a legal perspective, the adequacy of informed consent and the HCP's understanding of their duty of care are pivotal, yet such practices can themselves harm the patient-provider relationship (12). However, few studies have empirically examined whether participation in legal training actually correlates with a reduction in complaints, or how legal consciousness interacts with clinical behavior to influence outcomes.

Another gap in the existing literature is the exclusive focus on physicians. Nurses, who spend the most time with patients and often act as the primary bridge of communication, are equally susceptible to complaints and violence (13). Excluding nurses from risk assessment models provides an incomplete picture of the clinical risk landscape. A comprehensive risk management strategy requires a holistic view that encompasses the entire medical team.

To address these gaps, this study integrates medical, psychological, and legal perspectives to construct a novel predictive tool. We hypothesized that occupational burnout, heavy workload (specifically night shifts), and insufficient legal awareness are independent predictors of medical complaints. Using data from a tertiary hospital in Harbin, China, we aimed to: (1) identify key risk factors associated with patient complaints among both physicians and nurses; and (2) develop and validate a nomogram to predict the individual probability of incurring a medical complaint. This tool is intended to assist hospital administrators in early identification of high-risk staff and the implementation of targeted preventive interventions.

## 2 Methods

### 2.1 Study design and setting

This single-center, cross-sectional study was conducted at the Fourth Affiliated Hospital of Harbin Medical University, a large tertiary teaching hospital in Northeast China. The study period spanned from May 2024 to August 2025. The protocol was reviewed and approved by the Ethics Committee of the Fourth Affiliated Hospital of Harbin Medical University. All participants provided written informed consent prior to data collection. The study reporting adheres to the STROBE (Strengthening the Reporting of Observational Studies in Epidemiology) guidelines (14) and TRIPOD (Transparent Reporting of a multivariable prediction model for Individual Prognosis Or Diagnosis) statement (15).

### 2.2 Participants and data source

The study population consisted of healthcare professionals (HCPs), including physicians and nurses, working in clinical departments with historically high risks of medical disputes. The target departments included General Surgery (including Emergency Surgery), Orthopedics, Neurosurgery, Thoracic Surgery, Urology (Wards 1 and 2), Neurology (Ward 4 and others), Cardiology (Wards 1–8 and CCU), and the Endoscopy Center.

The inclusion criteria were: (1) holding a valid practicing certificate; (2) having worked in the current department for at least 6 months; and (3) volunteering to participate in the survey. Exclusion criteria were: (1) interns, refresher doctors/nurses, or rotating residents; (2) staff on long-term leave (e.g., maternity leave, sick leave) during the study period; and (3) questionnaires with missing data >10% or logical errors.

A total of 420 questionnaires were distributed. Of the 450 initially identified staff, 30 were not distributed questionnaires because 18 were on temporary leave during the survey period and 12 explicitly declined participation at the initial contact. After excluding 14 invalid responses, 406 valid questionnaires were included in the final analysis (effective response rate: 96.7%). The final sample comprised 187 physicians and 219 nurses, consistent with the staffing distribution of the selected departments.

### 2.3 Variables and measurements

Data were collected using a structured questionnaire designed for this study (see Supplementary material S1). To ensure data accuracy and minimize recall bias, objective workload data and complaint records were cross-verified with the hospital's Hospital Information System (HIS) and the Medical Disputes Office database using encrypted staff IDs.

#### 2.3.1 Demographic and occupational characteristics

Variables included age, gender, education level (College/Bachelor/Master/PhD), professional title (Physician: Resident, Attending, Associate Chief, Chief; Nurse: Nurse, Senior Nurse, Charge Nurse, Associate Chief Nurse, Chief Nurse), clinical tenure (years), and department.

#### 2.3.2 Workload indicators

Workload was assessed by: (1) average weekly working hours; (2) number of night shifts per month; and (3) daily patient load (average number of inpatients managed or nursing care provided per day). These were categorized into ordinal variables for analysis.

#### 2.3.3 Burnout assessment

The Maslach Burnout Inventory-Human Services Survey (MBI-HSS) (16) was adapted to evaluate professional burnout. We utilized the validated Chinese version of the MBI-HSS. It comprises three subscales: Emotional Exhaustion (EE), Depersonalization (DP), and Personal Accomplishment (PA). Items were scored on a 5-point Likert scale (1 = “Never” to 5 = “Every day”). High burnout was defined using standard threshold criteria: EE score ≥ 27, DP score ≥ 10, and PA score ≤ 33. Cronbach's alpha for the scale in this study was 0.89, indicating good internal consistency.

#### 2.3.4 Medicolegal factors

This dimension, specifically integrated for this study, included: (1) *Defensive Medicine Tendency*: Frequency of ordering unnecessary tests or avoiding high-risk procedures to prevent litigation (1 = “Never” to 4 = “Always”); (2) *Legal Training Frequency*: Number of hospital-organized legal training sessions attended in the past year (0, 1–2, 3–4, ≥5); and (3) *Legal Awareness*: Self-perceived understanding of core medical laws (e.g., the Physicians Law of the PRC).

#### 2.3.5 Outcome variable

The primary outcome was the occurrence of a “medical complaint” in the past 12 months. This was strictly defined as any formal written complaint filed by a patient or family member to the hospital administration, or a medical dispute requiring legal mediation or litigation. Informal verbal grievances were explicitly excluded from this definition. Data were binary (Yes/No).

### 2.4 Statistical analysis

Statistical analyses were performed using R software (version 4.2.3; The R Foundation for Statistical Computing). Specific R packages used included ‘glmnet‘ for LASSO, ‘rms‘ for nomogram construction, and ‘rmda‘ for decision curve analysis. Continuous variables obeying normal distribution were presented as mean ± standard deviation (SD) and compared using the Student's *t*-test; otherwise, median (IQR) and Mann-Whitney U test were used. Categorical variables were expressed as frequencies (%) and compared using the Chi-square test or Fisher's exact test.

To ensure model robustness and avoid overfitting, the Least Absolute Shrinkage and Selection Operator (LASSO) regression was initially applied for variable selection. The optimal tuning parameter (lambda) in the LASSO model was selected based on the minimum criteria (lambda.min) via 10-fold cross-validation. Features with non-zero coefficients in the LASSO analysis were included in the subsequent multivariable logistic regression model. Prior to multivariable logistic regression, multicollinearity was assessed using the Variance Inflation Factor (VIF); all selected variables exhibited a VIF < 2.0, indicating no significant multicollinearity. Odds ratios (ORs) and 95% confidence intervals (CIs) were calculated.

Based on the independent predictors identified, a nomogram was constructed to predict the probability of medical complaints. The model's performance was evaluated using the concordance index (C-index) for discrimination. Calibration was assessed using calibration plots with 1,000 bootstrap resamples. Internal validation was conducted using these 1,000 bootstrap resamples to calculate the optimism-corrected C-index. Decision Curve Analysis (DCA) was conducted to determine the clinical usefulness of the nomogram by quantifying the net benefits at different threshold probabilities.

## 3 Results

### 3.1 Baseline characteristics of the study population

**Figure 1:**
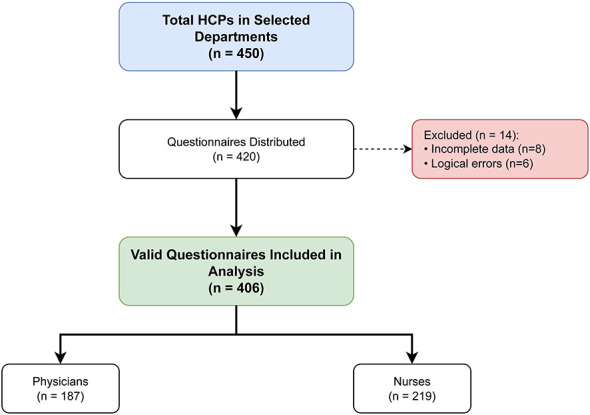
Flowchart of participant selection. The diagram illustrates the recruitment process of healthcare professionals from the Fourth Affiliated Hospital of Harbin Medical University. Of the 450 initially identified staff, 420 questionnaires were distributed to eligible physicians and nurses (30 were excluded due to temporary leave or refusal). Fourteen participants were excluded based on specific criteria (incomplete responses or logical errors), resulting in a final study population of 406 participants (187 physicians and 219 nurses) for the development and validation of the nomogram.

A total of 450 healthcare professionals were initially identified from the target departments. As previously noted, 30 individuals were excluded prior to distribution due to absence or refusal. Among them, 420 questionnaires were distributed. After excluding 14 participants due to incomplete data (*n* = 8) or logical errors (*n* = 6), a total of 406 valid questionnaires were included in the final analysis (effective response rate: 96.7%). The detailed screening and inclusion process is shown in Figure 1. To assess potential selection bias, we compared the basic demographics of the 406 participants with the 44 non-participants (including those who refused or had invalid data). As detailed in Supplementary Table S1, there were no significant differences in mean age (36.4 vs. 37.1 years, *P* = 0.542), gender distribution (females: 68.2% vs. 65.9%, *P* = 0.751), or department distribution (*P* = 0.428), suggesting minimal selection bias. The demographic and occupational characteristics of the 406 participants are summarized in Table 1. The study population included 187 physicians (46.1%) and 219 nurses (53.9%). The mean age was 36.4 ± 8.2 years, and females accounted for 68.2% of the sample (predominantly in the nursing group). In terms of education, 42.8% held a Master's degree or higher. Regarding workload, a substantial proportion (38.4%) reported working more than 50 h per week, and 29.1% undertook more than 5 night shifts per month.

**Table 1:** Baseline characteristics of healthcare professionals stratified by history of medical complaints.

Characteristic	Total (*N* = 406)	No complaint (*n* = 328)	Complaint (*n* = 78)	*P*-value
**Age (years), mean** **±SD**	36.4 ± 8.2	35.8 ± 7.9	38.9 ± 9.1	0.004^*^
Gender, *n* (%)				
Male	129 (31.8%)	100 (30.5%)	29 (37.2%)	0.215
Female	277 (68.2%)	228 (69.5%)	49 (62.8%)	
Role, *n* (%)				
Physician	187 (46.1%)	144 (43.9%)	43 (55.1%)	0.042^*^
Nurse	219 (53.9%)	184 (56.1%)	35 (44.9%)	
Department, *n* (%)				
Neurosurgery	26 (6.4%)	19 (5.8%)	7 (9.0%)	0.018^*^
General Surgery	23 (5.7%)	17 (5.2%)	6 (7.7%)	
Orthopedics	20 (4.9%)	14 (4.3%)	6 (7.7%)	
Cardiology	205 (50.5%)	170 (51.8%)	35 (44.9%)	
Others	132 (32.5%)	108 (32.9%)	24 (30.8%)	
Night Shifts (per month), *n* (%)				
0–2	150 (36.9%)	135 (41.2%)	15 (19.2%)	<0.001^*^
3–5	138 (34.0%)	110 (33.5%)	28 (35.9%)	
>5	118 (29.1%)	83 (25.3%)	35 (44.9%)	
Legal Training (times/year), *n* (%)				
≥ 3	160 (39.4%)	145 (44.2%)	15 (19.2%)	<0.001^*^
1–2	146 (36.0%)	116 (35.4%)	30 (38.5%)	
0	100 (24.6%)	67 (20.4%)	33 (42.3%)	
**Burnout: EE Score (mean** **±SD)**	24.5 ± 5.6	23.1 ± 5.1	29.8 ± 6.2	<0.001^*^
**Burnout: DP Score (mean** **±SD)**	12.3 ± 4.1	11.5 ± 3.8	15.6 ± 4.5	<0.001^*^

^*^indicates statistical significance (*P* < 0.05). EE, Emotional Exhaustion; DP, Depersonalization.

Of the total sample, 78 HCPs (19.2%) had experienced at least one formal medical complaint in the pre ceding year. The incidence varied significantly by department, with Neurosurgery (28.5%) and Emergency Surgery (26.1%) showing the highest rates, while the Endoscopy Center showed the lowest (4.3%).

### 3.2 Variable selection via LASSO regression

**Figure 2:**
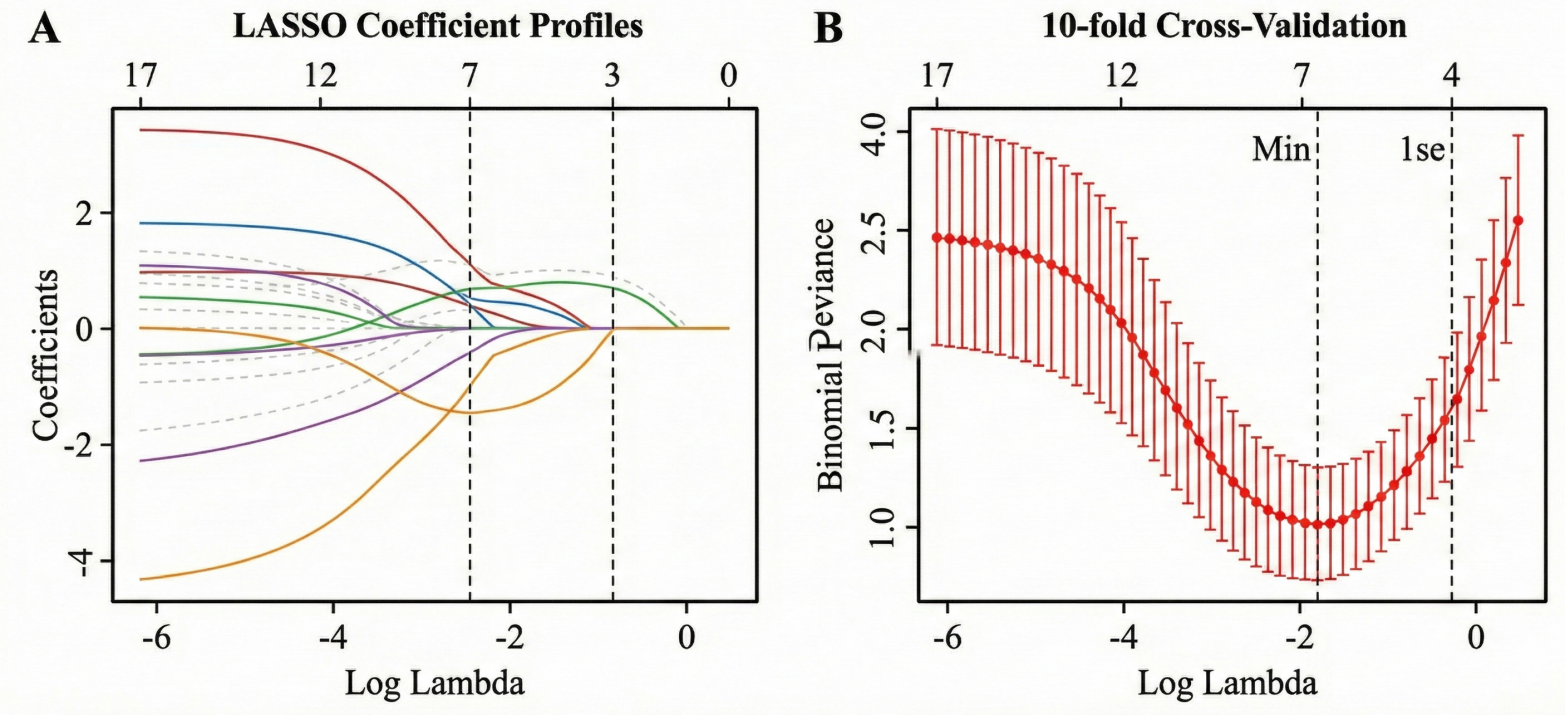
Variable selection using the LASSO binary logistic regression model. **(A)** LASSO coefficient profiles of the 18 candidate variables. Each curve corresponds to a variable. **(B)** Tuning parameter (lambda) selection in the LASSO model using 10-fold cross-validation. The dotted vertical lines represent the optimal values (lambda.min and lambda.1se) derived from the minimum criteria and the 1-standard error criteria.

A total of 18 potential predictors were entered into the LASSO regression model. Figure 2A illustrates the coefficient profiles of the variables, and Figure 2B shows the selection of the optimal lambda (λ) using 10-fold cross-validation. Seven variables with non-zero coefficients were selected for the subsequent analysis: Professional Title, Weekly Working Hours, Night Shifts, Emotional Exhaustion (EE) Score, Depersonalization (DP) Score, Defensive Medical Behavior, and Legal Training Frequency.

### 3.3 Identification of independent risk factors

Multivariable logistic regression analysis (Table 2) incorporating the LASSO-selected variables revealed four independent risk factors significantly associated with medical complaints. All variables included in the model showed VIF values below 2.0 (range 1.15–1.88), ruling out multicollinearity.

**Table 2:** Univariate and multivariate logistic regression analysis for risk factors of medical complaints.

Variable	Univariate analysis		Multivariate analysis	
	OR (95% CI)	*P*-value	OR (95% CI)	*P*-value
Age (>40 vs ≤ 40)	1.45 (0.98–2.12)	0.062	1.12 (0.68–1.85)	0.654
Role (Physician vs Nurse)	1.56 (1.02–2.38)	0.041^*^	1.34 (0.82–2.15)	0.245
Night Shifts				
0–2 (Ref)	1.00		1.00	
3-5	2.29 (1.25–4.35)	0.008^*^	1.45 (0.89–2.56)	0.125
>5	3.79 (2.10–7.15)	<0.001^*^	1.65 (1.12–2.43)	0.012^*^
Emotional Exhaustion (High)	2.10 (1.45–3.10)	<0.001^*^	1.85 (1.23–2.78)	0.003^*^
Depersonalization (High)	2.45 (1.68–3.65)	<0.001^*^	2.10 (1.45–3.05)	<0.001^*^
Legal Training				
≥3 (Ref)	1.00		1.00	
1–2	2.50 (1.35–4.80)	0.004^*^	1.85 (0.95–3.65)	0.072
0	4.75 (2.55–9.20)	<0.001^*^	2.45 (1.56–3.89)	<0.001^*^
Defensive Behavior	1.68 (1.10–2.56)	0.015^*^	1.42 (0.96–2.12)	0.082

OR, Odds Ratio; CI, Confidence Interval. Variables with *P* < 0.05 in univariate analysis were entered into the multivariate model.

^*^ indicates statistical significance *P* < 0.05.

First, **burnout** played a pivotal role: HCPs with high Emotional Exhaustion scores were nearly twice as likely to receive complaints (OR = 1.85, 95% CI: 1.23–2.78, *P* = 0.003), and those with high Depersonalization scores faced even greater risk (OR = 2.10, 95% CI: 1.45–3.05, *P* < 0.001).

Second, **workload** was a critical determinant. Staff performing >5 night shifts per month had a significantly higher risk compared to those with 0–2 shifts (OR = 1.65, 95% CI: 1.12–2.43, *P* = 0.012).

Third, **legal training** showed a protective effect. Compared to those who attended ≥3 training sessions per year, HCPs who attended 0–1 session had a markedly increased risk of disputes (OR = 2.45, 95% CI: 1.56–3.89, *P* < 0.001).

Interestingly, **Defensive Medical Behavior** exhibited a complex relationship; while “frequent” defensive behavior was associated with higher complaints in univariate analysis, it did not retain statistical significance in the multivariate model (*P* = 0.082), indicating that its effect was attenuated after adjusting for major confounders such as occupational burnout and workload.

### 3.4 Construction and validation of the nomogram

**Figure 3:**
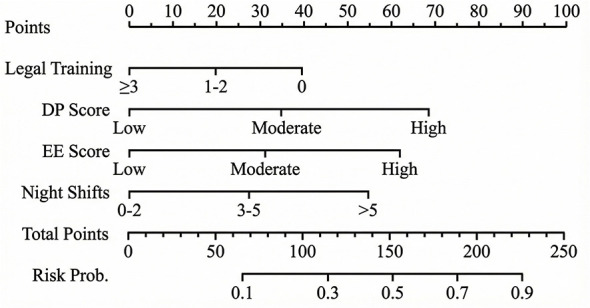
Nomogram for predicting the risk of medical complaints among healthcare professionals. The nomogram is used by summing the points identified on the points scale for each variable. The total points projected on the bottom scale indicate the predicted probability of experiencing a medical complaint in the past year. EE, Emotional Exhaustion; DP, Depersonalization.

Based on the multivariate logistic regression model, a nomogram was developed to predict the individual risk of medical complaints (Figure 3). Each predictor (Night Shifts, EE Score, DP Score, Legal Training) was assigned a point score on the top scale. The sum of these points corresponds to the “Total Points,” which can be projected onto the bottom scale to estimate the probability of a medical complaint.

**Figure 4:**
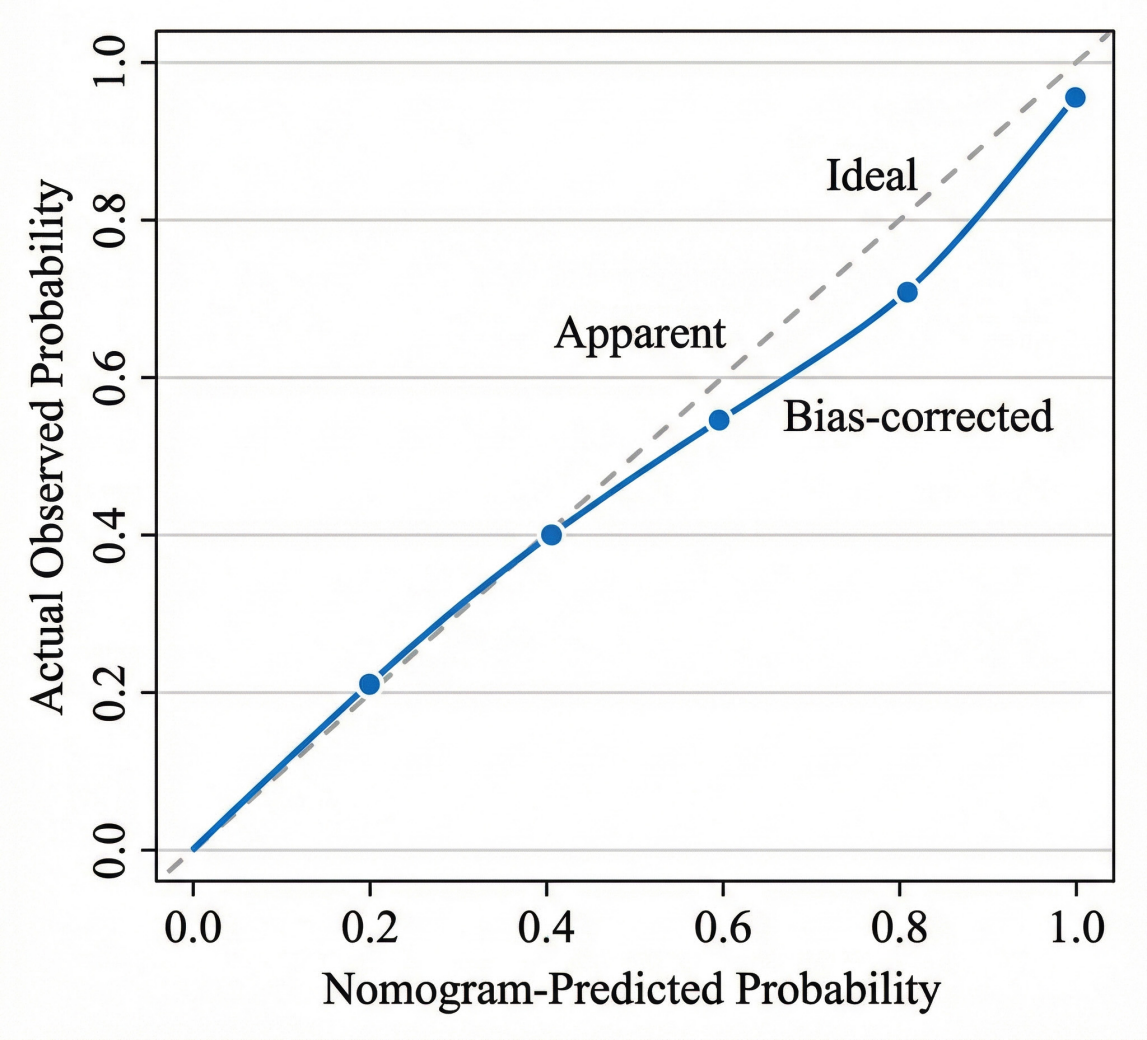
Calibration curve of the nomogram. The x-axis represents the predicted probability of medical complaints, and the y-axis represents the actual observed probability. The diagonal gray line represents a perfect prediction by an ideal model. The solid black line represents the performance of the nomogram, indicating a good fit between prediction and observation. Internal validation was performed via 1,000 bootstrap resamples.

The model demonstrated good discrimination, with a C-index of 0.82 (95% CI: 0.76–0.88), indicating a strong ability to distinguish between HCPs who would and would not face complaints. Internal validation via 1,000 bootstrap resamples yielded an optimism-corrected C-index of 0.811, confirming the model's stability and robustness. The calibration plot (Figure 4) revealed excellent agreement between the nomogram-predicted probabilities and actual observations, with the bias-corrected curve lying close to the ideal 45-degree line.

**Figure 5:**
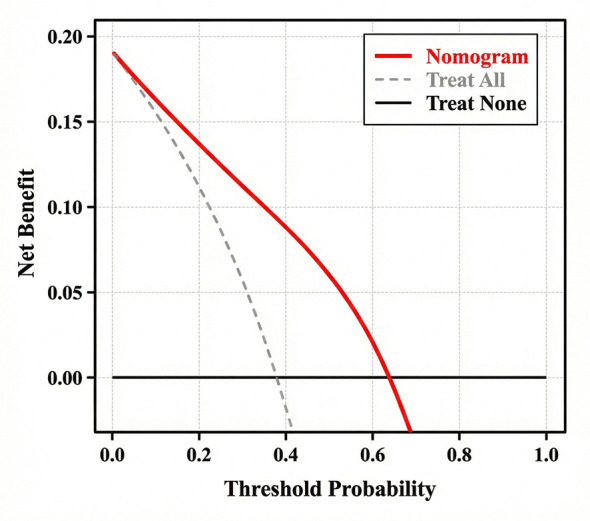
Decision Curve Analysis (DCA) of the nomogram. The y-axis measures the net benefit. The red line represents the nomogram. The gray line represents the assumption that all HCPs receive complaints (Treat All), and the black horizontal line represents the assumption that no HCPs receive complaints (Treat None). The decision curve shows that the nomogram adds more net benefit than either the “treat all” or “treat none” strategies for a threshold probability range of 10% to 80%.

Furthermore, Decision Curve Analysis (DCA) (Figure 5) showed that using this nomogram to screen for high-risk staff provides a greater net benefit than either the “treat-all” (assuming everyone is high risk) or “treat-none” strategies across a wide range of threshold probabilities (10% to 80%). This confirms the clinical and managerial utility of the model.

## 4 Discussion

In this single–center retrospective study, we successfully developed and validated a nomogram to predict the risk of medical complaints among healthcare professionals (HCPs). Our findings indicate that nearly one–fifth of the staff experienced complaints in the past year, a prevalence that aligns with recent reports of the substantial burden of formal complaints on healthcare workers (17). By integrating occupational burnout, workload, and legal training into a unified model, we provide a novel tool for hospital administrators to proactively identify high–risk individuals. The robust C–index (0.82) suggests that this model has high discriminative power.

### 4.1 The mechanism: from burnout to legal liability

Our study identified Emotional Exhaustion and Depersonalization as the strongest predictors of medical complaints. This finding is consistent with evidence linking depersonalization to a higher likelihood of self–reported medical errors and adverse patient–care outcomes (18). The relationship can be interpreted through the lens of Tort Law, specifically regarding the “Duty of Care.” In medical malpractice litigation, a breach of duty is often established not only by technical errors but also by failures in communication or lack of diligence, as seen in cases where misdiagnosis or delayed referral directly lead to legal claims (19). Burned–out professionals, characterized by cynicism and detachment (Depersonalization), are more likely to exhibit impaired emotional recognition and reduced empathy, which can manifest as “communication reactance” and leave patients feeling unheard or disrespected (9). When a patient perceives a lack of empathy, the threshold for filing a complaint lowers drastically, transforming minor clinical deviations into formal legal disputes. Thus, burnout is not merely an occupational health issue but a direct precursor to legal liability.

### 4.2 The “workload-risk” paradox and safety culture

Excessive night shifts (e.g., >5 per month) are independently associated with higher risks of complaints. This aligns with the “Fatigue-Error” hypothesis, where sleep deprivation impairs cognitive function and decision-making, leading to medical errors (20). Recent studies confirm a non-linear increase in adverse events with higher monthly night shift frequency (21). Beyond biological fatigue, this reflects a systemic failure in safety culture. Chronic understaffing forces healthcare professionals into perpetual crisis management, leaving little capacity for meticulous documentation and patient education essential for legal protection (22). Administrative interventions to cap night shift frequencies could therefore yield tangible reductions in medicolegal costs.

### 4.3 The legal training dilemma: quantity vs. quality

A striking protective effect is observed with frequent legal training; healthcare professionals attending ≥3 sessions annually show significantly lower complaint risks. This supports the efficacy of continuing medicolegal education, with studies demonstrating that targeted training, such as simulation-based workshops, increases physicians‘ comfort and preparedness in litigation scenarios (23). However, qualitative feedback suggests “Defensive Medicine” behaviors did not significantly reduce complaints in multivariate models. From a Law and Economics perspective, defensive medicine acts as an “insurance premium” paid in time and effort to avoid liability. Yet, evidence indicates this strategy may be inefficient and counterproductive, as excessive defensive behaviors (e.g., over-prescription of tests) increase patients' financial burden and anxiety, potentially triggering the very complaints they aim to prevent (24). Consequently, legal training should pivot from “how to hide errors” to “how to communicate risks and obtain valid Informed Consent.” True informed consent is not merely a signature on a form—which offers limited legal protection if the process is flawed—but a meaningful dialogue that aligns patient expectations with medical realities.

### 4.4 Differences between physicians and nurses

Although our nomogram pooled physicians and nurses to maximize statistical power, subgroup trends (Supplementary Table S2) indicated distinct risk profiles. Physicians were more vulnerable to disputes arising from treatment efficacy and surgical complications, as evidenced by studies focusing on litigation in surgical specialties (25). In contrast, nurses faced complaints largely related to service attitude and communication, which are consistently among the most common themes in patient feedback (26), as well as challenges related to procedural timeliness (27). This dichotomy highlights the need for role-specific risk management strategies: technical and decision-making support for doctors, and emotional labor support and communication skills training for nurses.

### 4.5 Clinical implications

The proposed nomogram transforms abstract risk factors into a visible, calculable probability, aligning with the growing use of predictive models for individualized risk assessment in healthcare professionals (28). Hospital managers can use this tool for regular screening. For staff identified as “high risk” (e.g., probability >50%), immediate interventions such as mandatory leave, psychological counseling, or intensive communication workshops can be deployed. This moves risk management from a reactive “post-event punishment” model to a proactive “pre-event prevention” model, leveraging data-driven tools for early identification and mitigation.

### 4.6 Limitations

First, as a single-center study without an independent external validation cohort, the generalizability of the nomogram to other regions or hospital tiers remains to be verified. Future multicenter studies are required to externally validate and refine the model before it can be implemented as a widespread practical screening tool. Second, the cross-sectional design precludes causal inference; for instance, it is possible that receiving a complaint causes burnout, rather than vice versa (reverse causality). Third, the outcome variable was self-reported initially, which might introduce recall bias or social desirability bias, although we attempted to mitigate this by cross-referencing with administrative data where possible. Fourth, participation was voluntary. HCPs with severe burnout or recent complaints might have declined to participate due to fatigue or fear of consequences. Although our comparison of basic demographics between participants and non-participants showed no significant differences, this self-selection could still lead to an underestimation of the true incidence of complaints. Fifth, several potential confounding factors, such as department-level staffing ratios, specific patient case complexity, and patient volume per clinician, were not included in our model and warrant future investigation.

## 5 Conclusion

In conclusion, this study demonstrates that HCP burnout, excessive workload, and insufficient legal training are potent predictors of medical dispute risks. We successfully constructed and validated a nomogram that integrates these factors to predict the individual probability of facing complaints. This tool underscores the critical need for healthcare institutions to address staff wellbeing and legal literacy not just as HR benefits, but as essential components of patient safety and legal risk management strategies.

## Data Availability

The original contributions presented in the study are included in the article/Supplementary material, further inquiries can be directed to the corresponding author.

## Supplementary material

Supplementary Figure S1 Correlation heatmap. This heatmap visualizes the Spearman correlation coefficients between Burnout dimensions (EE, DP, PA) and Defensive Medical Behaviors. Red indicates a positive correlation, while blue indicates a negative correlation. Significant positive correlations were observed between EE/DP scores and defensive behaviors.
