# Supplementary material for: Construction and validation of nomogram model for high-risk early warning of medical complaints based on occupational characteristics and workload of medical staff
Source: Front Public Health. 2026 May 21;14:1816281. doi: 10.3389/fpubh.2026.1816281 (PMC13235659; doi:10.3389/fpubh.2026.1816281)
Supplement: Supplementary file 1 [file Table_1.docx]

**Supplementary Table S1. Comparison of basic characteristics between participating and non-participating healthcare professionals.**

| **Characteristic** | **Participants (n=406)** | **Non-participants (n=44)** | **P-value** |
| --- | --- | --- | --- |
| Age (years), mean ± SD | 36.4 ± 8.2 | 37.1 ± 8.5 | 0.542 |
| Gender, n (%) |  |  | 0.751 |
| Male | 129 (31.8%) | 15 (34.1%) |  |
| Female | 277 (68.2%) | 29 (65.9%) |  |
| Department, n (%) |  |  | 0.428 |
| Cardiology | 205 (50.5%) | 18 (40.9%) |  |
| Surgical Departments* | 69 (17.0%) | 10 (22.7%) |  |
| Others | 132 (32.5%) | 16 (36.4%) |  |

Note: *Surgical departments include Neurosurgery, General Surgery, and Orthopedics. Data for non-participants were extracted from the hospital administrative system. No statistically significant differences were observed between the two groups, indicating minimal selection bias.
