# Supplementary material for: Construction and validation of nomogram model for high-risk early warning of medical complaints based on occupational characteristics and workload of medical staff
Source: Front Public Health. 2026 May 21;14:1816281. doi: 10.3389/fpubh.2026.1816281 (PMC13235659; doi:10.3389/fpubh.2026.1816281)
Supplement: Supplementary file 2 [file Table_2.docx]

**Supplementary Table S2. Subgroup Analysis of Risk Factors Stratified by Professional Role (Physician vs. Nurse).**

| **Variable** | **Physicians (n=187)** | | | **Nurses (n=219)** | | |
| --- | --- | --- | --- | --- | --- | --- |
|  | **Complaint (%)** | **OR (95% CI)** | **P-value** | **Complaint (%)** | **OR (95% CI)** | **P-value** |
| **High Burnout (EE)** | 32 (68.1%) | 2.10 (1.15–3.85) | 0.015* | 28 (60.9%) | 1.65 (0.92–2.95) | 0.092 |
| **High Burnout (DP)** | 35 (74.5%) | 2.45 (1.30–4.55) | 0.005* | 30 (65.2%) | 1.88 (1.05–3.40) | 0.034* |
| **Night Shifts (>5)** | 15 (31.9%) | 1.25 (0.65–2.40) | 0.502 | 30 (65.2%) | 2.15 (1.20–3.85) | 0.010* |
| **Legal Training (0)** | 25 (53.2%) | 2.85 (1.50–5.45) | 0.001* | 18 (39.1%) | 2.05 (1.10–3.80) | 0.024* |

Note: * P < 0.05. EE: Emotional Exhaustion; DP: Depersonalization. Physicians appeared more sensitive to burnout-related risks, while nurses were more affected by shift workload.
