# Supplementary material for: Construction and validation of nomogram model for high-risk early warning of medical complaints based on occupational characteristics and workload of medical staff
Source: Front Public Health. 2026 May 21;14:1816281. doi: 10.3389/fpubh.2026.1816281 (PMC13235659; doi:10.3389/fpubh.2026.1816281)
Supplement: Supplementary file 3 [file Data_Sheet_1.pdf]

Questionnaire on Occupational Risk and Workload of Healthcare Professionals

Data Collection Unit: The Fourth Affiliated Hospital of Harbin Medical University

---

Dear Colleagues:

Greetings! This is an interdisciplinary study concerning the "Occupational Characteristics, Legal Awareness, and Medical Complaint Risk of Healthcare Professionals." Your truthful feedback will help us build a scientific early warning model, providing a basis for reducing occupational risks and improving doctor-patient relationships. This questionnaire is strictly ***anonymous****, and data will be used solely for academic statistical purposes.*

Part I: Basic Information and Job Characteristics

1. Your Employee ID (Optional, only for matching dispute records in the backend):

____

2. Your Job Category:

○ Physician (Please fill in Q3) ○ Nurse (Please fill in Q4)

3. [For Physicians] Your Professional Title:

○ Resident ○ Attending Physician ○ Associate Chief Physician ○ Chief Physician

4. [For Nurses] Your Professional Title:

○ Nurse ○ Senior Nurse ○ Charge Nurse ○ Associate Chief Nurse
○ Chief Nurse

5. Your Department:

○ General Surgery ○ Orthopedics ○ Neurosurgery ○ Thoracic Surgery
○ Urology ○ Neurology ○ Cardiology ○ Others

6. Your Gender:

○ Male ○ Female

7. Your Age:

____________________ years old

8. Your Highest Education Level:

○ College or below ○ Bachelor ○ Master ○ PhD

9. Years of Clinical Practice:

____________________ years

Part II: Workload and Intensity

10. Your average weekly actual working hours (including overtime, shifts, paperwork, etc.):

○ < 40 hours ○ 40-50 hours ○ 51-60 hours ○ > 60 hours

11. Number of night shifts in the past month:

____

Part III: Legal Awareness and Training Status

12. In the past year, how many times have you participated in training related to "medical laws and regulations" or "prevention of medical disputes" organized by the hospital or department?

○ 0 times ○ 1-2 times ○ 3-4 times ○ 5 times or more

13. How would you rate your mastery of core medical laws and regulations such as the "Physicians Law" and the "Regulations on Prevention and Handling of Medical Disputes"?

○ Very unfamiliar ○ Only superficial knowledge ○ Basic mastery ○ Very familiar

14. Do you think the current legal training actually helps reduce your occupational risk?

○ No help at all, mere formality ○ Little help ○ Some help ○ Very helpful

Part IV: Occupational Burnout and Risk Perception

Please choose the option that best fits your true feelings over the **past month**:

Item Description	Never	Rarely	Sometimes	Often	Every day
15. I feel emotionally drained from my work (Emotional Exhaustion).	○	○	○	○	○
16. I treat some patients as if they were impersonal objects (Depersonalization).	○	○	○	○	○
17. I feel I cannot effectively solve the problems that arise in my work (Low Personal Accomplishment).	○	○	○	○	○

18. In your daily work, do you adopt "defensive medical behaviors" to avoid legal risks?

(e.g., doctors ordering extra tests/avoiding high-risk surgeries; nurses adding defensive nursing records, etc.)

○ Never ○ Occasionally ○ Often ○ Always

19. In the past year, have you experienced complaints or disputes from patients/families?

○ No ○ Yes, only verbal dissatisfaction ○ Yes, formal written complaint or dispute

---

This questionnaire is for academic research use only.
