# Supplementary figures and images for: Construction and validation of nomogram model for high-risk early warning of medical complaints based on occupational characteristics and workload of medical staff
Source: Front Public Health. 2026 May 21;14:1816281. doi: 10.3389/fpubh.2026.1816281 (PMC13235659; doi:10.3389/fpubh.2026.1816281)

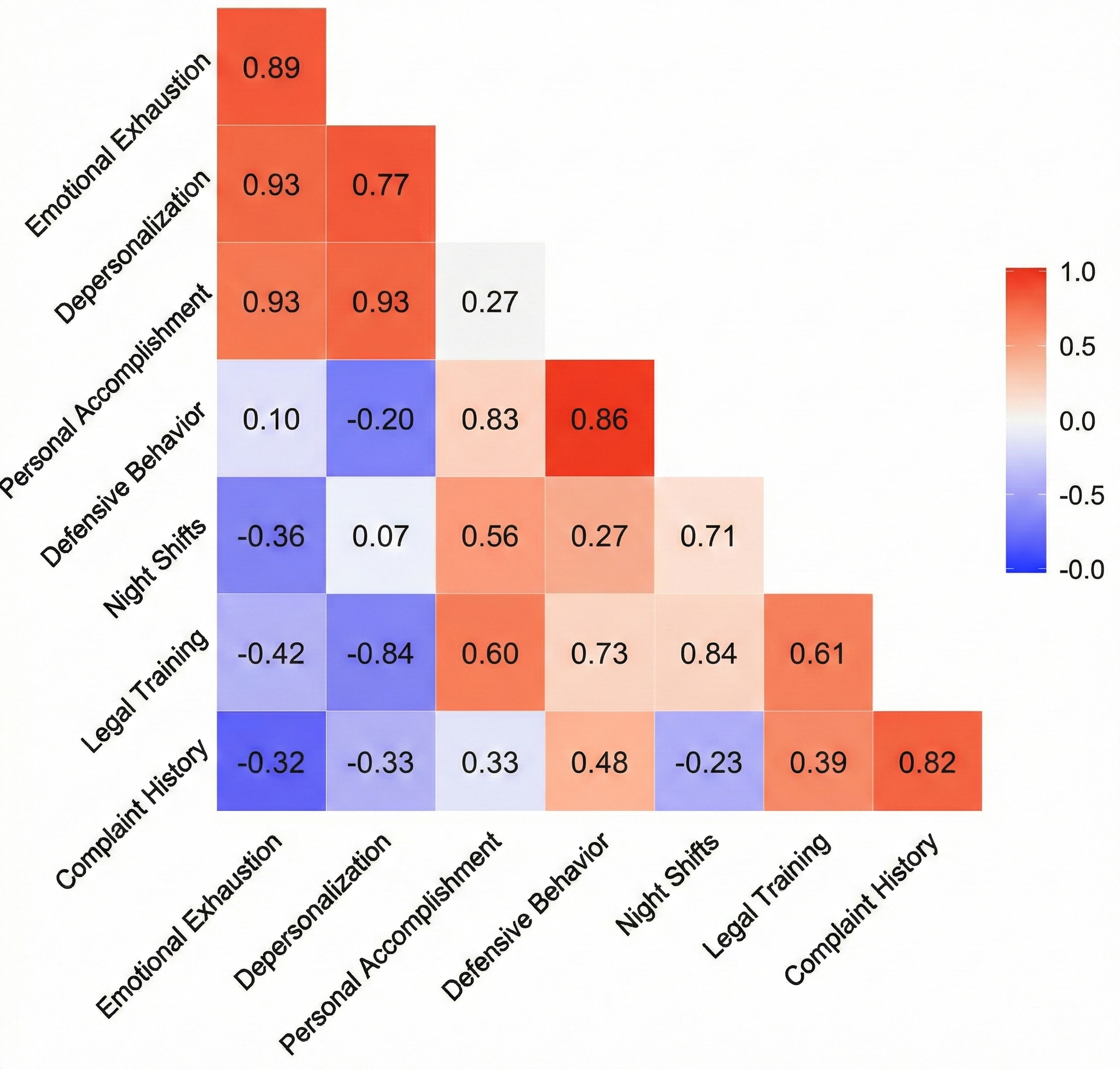

Supplement: Supplementary Figure S1 — Correlation heatmap. This heatmap visualizes the Spearman correlation coefficients between Burnout dimensions (EE, DP, PA) and Defensive Medical Behaviors. Red indicates a positive correlation, while blue indicates a negative correlation. Significant positive correlations were observed between EE/DP scores and defensive behaviors. [file Image_1.jpeg]
